# Pretreatment anxious depression as a predictor of side effect frequency and severity in escitalopram and aripiprazole adjunctive therapy

**DOI:** 10.1002/brb3.2555

**Published:** 2022-03-25

**Authors:** Caroline W. Espinola, Yuelee Khoo, Roohie Parmar, Ilya Demchenko, Benicio N. Frey, Roumen V. Milev, Arun V. Ravindran, Sagar V. Parikh, Keith Ho, Susan Rotzinger, Wendy Lou, Raymond W. Lam, Sidney H. Kennedy, Venkat Bhat, the CAN‐BIND Investigator Team

**Affiliations:** ^1^ Interventional Psychiatry Program St. Michael's Hospital Toronto Ontario Canada; ^2^ Department of Psychiatry University of Toronto Toronto Ontario Canada; ^3^ Department of Psychiatry and Behavioural Neurosciences McMaster University Hamilton Ontario Canada; ^4^ Mood Disorders Program and Women's Health Concerns Clinic St. Joseph's Healthcare Hamilton Ontario Canada; ^5^ Departments of Psychiatry and Psychology Queen's University Providence Care Kingston Ontario Canada; ^6^ Institute of Medical Science University of Toronto Toronto Ontario Canada; ^7^ Department of Psychiatry University of Michigan Ann Arbor Michigan USA; ^8^ Division of Biostatistics, Dalla Lana School of Public Health University of Toronto Toronto Ontario Canada; ^9^ Department of Psychiatry University of British Columbia Vancouver British Columbia Canada; ^10^ Li Ka Shing Knowledge Institute St. Michael's Hospital & Krembil Research Institute University Health Network Toronto Ontario Canada

**Keywords:** aripiprazole, depressive disorder, drug‐related effects and adverse reactions, escitalopram, serotonin uptake inhibitors

## Abstract

**Objective:**

To report side effect frequency and severity in patients with major depressive disorder (MDD) receiving escitalopram and aripiprazole adjunctive therapy and to examine whether pretreatment anxious depression is associated with the number and presence of specific side effects.

**Methods:**

188 of the 211 trial participants provided information on side effects during treatment with escitalopram (10–20 mg) for 8 weeks, and nonresponders received further augmentation on aripiprazole (2–10 mg) adjunctive therapy for another 8 weeks, whereas responders remained on escitalopram. Participants completed the Toronto Side Effects Scale at weeks 2, 4, 10, and 12. Covariate‐adjusted negative binomial regression and Wilcoxon tests examined the association between anxious depression (GAD‐7 ≥ 10) and number of side effects. Covariate‐adjusted logistic regression and chi‐square tests explored the association between anxious depression and specific side effects.

**Results:**

For both therapies, the most frequent side effects were also the most severe. They mostly related to the central nervous system (CNS) (i.e., drowsiness and nervousness). Between baseline and week 2, the number of side effects participants experienced (incidence rate ratio [IRR] = 1.38, *p* = .010) or had trouble with (IRR = 1.34, *p* = .026) was significantly higher among those with anxious depression for escitalopram but not adjunctive aripiprazole. Further, odds of experiencing and having trouble with nervousness and agitation were also significantly higher in anxious depression for escitalopram only (*p *< .05).

**Conclusion:**

Patients on escitalopram and aripiprazole adjunctive therapy may experience and have trouble with CNS side effects. Pretreatment anxious depression may predispose escitalopram recipients with MDD to developing side effects, especially those related to anxiety.

## Abstract 2

This study demonstrates the utility of a standardized side effects questionnaire (TSES) and advances our understanding of the association between pretreatment anxious depression and an increased number of side effects. Patients with anxious depression have significantly more side effects in the first weeks of escitalopram therapy than patients with nonanxious depression, particularly those related to anxiety (i.e., nervousness, agitation).

## 1 INTRODUCTION

Major depressive disorder (MDD) is a highly prevalent psychiatric disorder worldwide and frequently cooccurs with anxiety symptoms, a condition known as anxious depression (Ferrari et al., 2013; Smith, 2014). In the Sequenced Treatment Alternatives to Relieve Depression (STAR*D) study, 53% of its 2876 patients with MDD had anxious depression, defined using a ≥7 score on the Hamilton Depression Rating Scale's (HAM‐D) Anxiety/Somatization factor (ASF) (Fava et al., 2008). Another review found the prevalence of anxious depression to range between 54 and 78% using the DSM‐5 specifier, major depressive episode with anxious distress (Gaspersz et al., 2018).

As a frontline treatment, escitalopram, a selective serotonin reuptake inhibitor (SSRI), is widely prescribed since it has high treatment efficacy in MDD (Cipriani et al., 2018; Kennedy et al., 2006; Kennedy et al., 2009; Wade et al., 2006; Wade et al., 2002) and anxious depression (Bandelow et al., 2007). Among patients with MDD who are not initially responsive to escitalopram or other antidepressants, aripiprazole adjunctive therapy has also shown to be more effective than adjunctive placebo in reducing the severity of depressive symptoms in MDD and anxious depression (Berman et al., 2009; Berman et al., 2011; Marcus et al., 2008; Trivedi et al., 2008). However, both escitalopram and adjunctive aripiprazole are associated with side effects (Baldwin et al., 2007; Berman et al., 2009; Berman et al., 2011; Burke, 2002; Höschl & Svestka, 2008; Marcus et al., 2008). For instance, evidence shows that about 60% of patients on antidepressant therapy experience at least one side effect (Sanchez et al., 2014) and adjunctive aripiprazole may frequently lead to side effects (e.g., akathisia and fatigue) (Marcus et al., 2008). Although these symptoms tend to be mild and temporary, discontinuation rates due to adverse events may be as high as 10% in patients using escitalopram (Pastoor & Gobburu, 2014). In order to optimize patient monitoring and promote adherence, it is thus important to identify factors that predispose patients with MDD to developing these side effects.

A potential factor is anxious depression given its high prevalence in MDD. Findings were conflicting. A STAR*D study found that citalopram recipients with pretreatment anxious depression (defined using the HAM‐D ASF) had side effects that were greater in frequency, intensity/severity, and burden than those without anxious depression (Fava et al., 2008). On the other hand, a study on escitalopram and paroxetine found that anxious depression (defined using a ≥20 score on the Hamilton Anxiety Rating Scale [HAM‐A]) was not associated with a higher frequency of individual side effects and a higher proportion of participants with side effects (Boulenger et al., 2010). Another study on escitalopram and duloxetine also found that those with anxious depression (defined using HAM‐A) did not have a higher mean number of side effects (Polychroniou et al., 2018). Similarly, a study on adjunctive aripiprazole did not find a significantly different side effect profile between participants with and without anxious depression (defined using HAM‐D ASF) (Trivedi et al., 2008).

These discrepant findings may be due to the varying methods used to collect data on side effects. The STAR*D study (Fava et al., 2008) used a combination of standardized side effect scales administered at every study visit (the Patient Rated Inventory of Side Effects and the Frequency, Intensity, and Burden of Side Effects Rating scale), whereas the other aforementioned studies (Boulenger et al., 2010; Polychroniou et al., 2018; Trivedi et al., 2008) relied on spontaneous reporting, which has been associated with side effects being underreported (Hazell & Shakir, 2006). Given this potential risk of underreporting side effects in previous studies using spontaneous reporting, there is a need for studies to use standardized side effect scales to reexamine the associations between anxious depression and side effect frequency and severity.

Since no studies, to our knowledge, have utilized standardized side effects scales to examine such associations in escitalopram and adjunctive aripiprazole, the present study utilized the Toronto Side Effects Scale (TSES) and examined a cohort of MDD outpatient participants who received escitalopram (10–20 mg/d) for 8 weeks (Phase I) and, for a second 8 weeks (Phase II), either remained on escitalopram or switched to aripiprazole adjunctive therapy (2–10 mg/d) based on their response status. There were two objectives: (i) assess the frequency and severity of side effects experienced in Phase I and Phase II; (ii) assess the association between pretreatment anxious depression and the number and presence of specific side effects in Phase I and Phase II. Based on available literature on escitalopram and adjunctive aripiprazole that used spontaneous reporting, we hypothesized that pretreatment anxious depression would not be associated to the number and presence of side effects a patient would experience (Boulenger et al., 2010; Polychroniou et al., 2018; Trivedi et al., 2008).

## 2 MATERIALS AND METHODS

### 2.1 Study design and participants

Data from the initial Canadian Biomarker Integration Network in Depression trial were used in a secondary analysis (CAN‐BIND‐1; ClinicalTrials.gov Identifier: NCT01655706). Adults 18–60 years of age with a current major depressive episode duration of ≥3 months and a MADRS score of ≥24 were prospectively recruited via community advertising and referral networks across six clinical centers. Ethics approval was obtained from the Research Ethics Board of each participating institution. The complete trial design and inclusion/exclusion criteria have been described elsewhere (Kennedy et al., 2019; Lam et al., 2016).

### 2.2 Measures

Data from clinician‐rated and patient‐reported outcome measures were collected via electronic data capture systems. They were then entered into a federated database Brain‐CODE for secure storage. The complete list of procedures, assessments (including clinical interviews, questionnaires, blood testing for drug levels), and their schedules were presented elsewhere (Kennedy et al., 2019; Lam et al., 2016).

**Significant Outcomes**

1. Central nervous system side effects are most frequent and severe for escitalopram and adjunctive aripiprazole.
2. Incidence rate of escitalopram side effects is higher in anxious depression.
3. Odds of nervousness and agitation side effects with escitalopram is higher in anxious depression.

**Limitations**

1. Trial lacked placebo group.
2. Sample size was small for the adjunctive aripiprazole treatment group.

### 2.3 Side effects

The TSES was used to assess central nervous system (CNS), gastrointestinal, and sexual side effects (Vanderkooy et al., 2002). Patients were asked by clinicians to indicate if they had experienced symptoms within the last 2 weeks, compared with the 2 weeks before starting escitalopram. Clinicians then rated the frequency and severity of 32 side effects using 5‐point Likert scales ranging from 1 (*Never*) to 5 (*Everyday*), and from 1 (*No Trouble*) to 5 (*Extreme Trouble*), respectively. There were three side effect items related to ejaculation and hence were male specific, whereas one “other” item allowed patients to specify a side effect not mentioned on the scale. The “other” item was not used in the present study. TSES was administered at weeks 2, 4, 10, and 12, primarily measuring side effects that occurred in the first and second 2‐week intervals of Phase 1 and Phase 2.

### 2.4 Depression and anxiety symptom severity

Depression symptom severity was assessed using the overall scores of MADRS, a 10‐item clinician‐rated scale (Montgomery & Asberg, 1979). Responders were defined as participants who experienced a ≥50% decline from baseline to week 8. For the present study, MADRS data at baseline and week 8 were utilized.

Anxiety symptom severity was assessed using the Generalized Anxiety Disorder 7‐item scale (GAD‐7) (Spitzer et al., 2006), a patient‐reported outcome measure. Minimal, mild, moderate, and severe anxiety were characterized with an overall score of 0–4, 5–9, 10–14, and 15–21, respectively. A score ≥10 has been used to identify anxiety severity that demands clinical attention. For the present study, we used this cut‐off to define anxious depression. GAD‐7 data at baseline and week 8 were utilized.

### 2.5 Statistical analysis

All participants with detectable blood levels of escitalopram and available side effect data at week 2 were included in the present analysis. The steps outlined below were performed for the TSES scores collected at weeks 2, 4, 10, and 12. Due to a prominent floor effect in the distribution of the scores, we dichotomized both frequency (“no symptoms” vs. “experienced symptoms”) and severity (“no trouble” vs. “had trouble”) variables according to the scores of 1 or >1. We then ranked the most frequent and severe side effects using the proportion of respondents in the “experienced symptoms” and “had trouble” categories. For each participant, we also counted the number of non‐male‐specific side effects one experienced or had trouble with.

To examine whether baseline anxious depression was associated with the number of side effects one experienced or had trouble with in the first (baseline to week 2) and second (weeks 2 to 4) 2‐week intervals, we utilized negative binomial regression (shape parameter set at 1), which is suitable for overdispersed count data (i.e., mean unequal to variance). Model fit was examined using the goodness‐of‐fit chi‐square test. Anxious depression status at baseline was the predictor variable and the number of side effects was the outcome variable. Baseline depression severity, age, and sex were included as covariates.

We then explored the association between baseline anxious depression and experiencing or having trouble with specific side effects. Chi‐square and Fisher's exact tests were first conducted on the top 10 most frequent and severe side effects in each 2‐week interval. Where a significant association existed, we further conducted logistic regression analysis adjusting for baseline depression severity, age, and sex.

As an exploratory analysis, we also ranked the frequency and severity of side effects in the third (weeks 8–10) and fourth (weeks 10–12) 2‐week intervals, for both participants who switched to aripiprazole adjunctive therapy and those who remained on escitalopram. For both cohorts, we conducted Wilcoxon rank‐sum tests to assess the difference between participants with and without anxious depression at week 8 in the number of side effects one experienced or had trouble with at both time intervals. We further explored the relationship between week 8 anxious depression status and experiencing or having trouble with specific side effects using the chi‐square or the Fisher's exact test.

Missing data was treated with complete case analysis. For 2‐week interval, multiple testing in chi‐square and Fisher's tests were adjusted for using the false discovery rate method (10 tests) (Benjamini & Hochberg, 1995). All other analyses were conducted with a two‐sided alpha level of 0.05 on R software (version 4.0.3) (R Core Team 2020).

## 3 RESULTS

### 3.1 Sociodemographic and clinical characteristics

Two hundred and eleven participants were enrolled in the CANBIND‐1 study at baseline. Three participants with undetectable plasma levels of escitalopram and 20 participants without side effect data were excluded. The final sample for analysis contained 188 participants (62% female) who completed TSES at week 2 (mean age = 35.1, SD = 12.6). The respective sample sizes for weeks 4, 10, and 12 were 185, 166, and 164 participants. Other sociodemographic and clinical characteristics are shown in Table 1. There were no significant differences in the baseline characteristics between our sample and excluded participants, with the exception of sex, where our sample in the trial had a significantly smaller proportion of females (62% vs. 75%; *p* = .002).

**TABLE 1:** Baseline sociodemographic and clinical characteristics

	Sample with side effect data (*n* = 188)			Sample without side effect data (*n* = 20)			Test of difference
Variables	Missing/unknown	*n*	%	Missing/unknown	*n*	%	*p* ^a^
Sex—females	0 (0%)	116	61.7%	0 (0%)	15	75.0%	.002^†^
Married/cohabitating	0 (0%)	51	27.1%	0 (0%)	4	20.0%	.492^†^
Employed/student	1 (0.5%)	120	63.8%	0 (0%)	11	55.0%	.419^†^
Current episode duration—<12 Months	10 (5.3%)	95	53.4%	4 (20%)	9	45.0%	.829^†^
Prior antidepressant treatment for current episode							.532^‡^
None	0 (0%)	108	57.4%	0 (0%)	13	65.0%	
No adequate	0 (0%)	30	16.0%	0 (0%)	3	15.0%	
1 adequate	0 (0%)	46	24.5%	0 (0%)	3	15.0%	
2 adequate	0 (0%)	4	2.1%	0 (0%)	1	5.0%	

	Missing/unknown	Mean	SD	Missing/unknown	Mean	SD	*p*
Age	0 (0%)	35.1	12.6	0 (0%)	35.4	12.0	.967^§^
Years of education	2 (1.1%)	16.9	2.2	0 (0%)	17.2	1.8	.693^§^
Age of MDD onset	7 (3.7%)	22.0	11.9	1 (5.0%)	22.0	11.9	.995^§^
Number of previous episodes	12 (6.4%)	5.0	8.4	2 (10.0%)	5.0	8.4	.832^§^
MADRS total score	0 (0%)	30.0	5.5	0 (0%)	28.7	6.4	.302^§^

Abbreviations: MADRS, Montgomery‐Asberg Depression Rating Scale; MDD, major depressive disorder.

^a^ Statistical significance for all tests were set at *p* < .05.

^†^ Chi‐square test.

^‡^ Fisher's exact test.

^§^ Wilcoxon‐rank sum test.

### 3.2 Escitalopram side effects: baseline to week 4

The five most frequent (F) and severe (S) side effects overlapped with each other in the first and second 2‐week intervals (Table 2). They were mostly related to CNS, gastrointestinal, and sexual functioning. From baseline to week 2, the five most common side effects were drowsiness, nausea, headache, weakness fatigue, and nervousness, respectively, both among AD and non‐AD participants. From week 2 to week 4, decreased libido appears in the top 5, whereas nausea prevalence decreased dramatically and is not included in the 10 most frequent and severe side effects due to escitalopram use.

**TABLE 2:** Top 10 most frequent and severe escitalopram side effects at between baseline to week 4

Baseline to week 2 frequency—have symptoms *n* (%)	Overall (*n* = 188)	No AD (*n* = 69)	AD (*n* = 119)	*p* *	Baseline to week 2 severity—have trouble *n* (%)	Overall (*n* = 188)	No AD (*n* = 69)	AD (n = 119)	*p* ^*^
Drowsiness	85 (45.2)	26 (37.7)	59 (49.6)	.347	Drowsiness	73 (38.8)	22 (31.9)	51 (42.9)	.420
Nausea	76 (40.4)	27 (39.1)	49 (41.2)	1.000	Nausea	69 (36.7)	24 (34.8)	45 (37.8)	.898
Headache	67 (35.6)	21 (30.4)	46 (38.7)	.583	Headache	58 (30.9)	17 (24.6)	41 (34.5)	.658
Weakness fatigue	60 (31.9)	19 (27.5)	41 (34.5)	.590	Weakness fatigue	53 (28.2)	18 (26.1)	35 (29.4)	.900
Nervousness	56 (29.8)	11 (15.9)	45 (37.8)	.027	Nervousness	49 (26.1)	9 (13.0)	40 (33.6)	.035
Dyspepsia	51 (27.1)	19 (27.5)	32 (26.9)	1.000	Agitation	47 (25.0)	10 (14.5)	37 (31.1)	.092
Agitation	51 (27.1)	11 (15.9)	40 (33.6)	.070	Dyspepsia	46 (24.5)	17 (24.6)	29 (24.4)	1.000
Dry mouth	47 (25.0)	12 (17.4)	35 (29.4)	.323	Dry MOUTH	41 (21.8)	10 (14.5)	31 (26.1)	.319
Decreased appetite	45 (23.9)	16 (23.2)	29 (24.4)	1.000	Decreased sleep	37 (19.7)	12 (17.4)	25 (21.0)	.898
Increased sleep	42 (22.3)	14 (20.3)	28 (23.5)	.583	Decreased appetite	33 (17.6)	11 (15.9)	22 (18.5)	.898

Weeks 2 to 4 frequency—have symptoms *n* (%)	Overall (*n* = 185)	No AD (*n* = 69)	AD (*n* = 116)	*p* *	Weeks 2 to 4 severity—have trouble *n* (%)	Overall (*n* = 185)	No AD (*n* = 69)	AD (n = 116)	*p* ^*^
Drowsiness	80 (43.2)	29 (42.0)	51 (44.0)	1.000	Drowsiness	71 (38.4)	25 (36.2)	46 (39.7)	.873
Weakness Fatigue	65 (35.1)	24 (34.8)	41 (35.3)	1.000	Weakness Fatigue	62 (33.5)	24 (34.8)	38 (32.8)	.904
Nervousness	54 (29.2)	10 (14.5)	44 (37.9)	.013	Nervousness	47 (25.4)	7 (10.1)	40 (34.5)	.005
Decreased Libido	50 (27.0)	17 (24.6)	33 (28.4)	.921	Headache	46 (24.9)	13 (18.8)	33 (28.4)	.405
Headache	49 (26.5)	15 (21.7)	34 (29.3)	.583	Decreased Libido	44 (23.8)	15 (21.7)	29 (25.0)	.873
Increased Sleep	46 (24.9)	14 (20.3)	32 (27.6)	.583	Agitation	40 (21.6)	7 (10.1)	33 (28.4)	.031
Agitation	45 (24.3)	9 (13.0)	36 (31.0)	.049	Anorgasmia	38 (20.5)	18 (26.1)	20 (17.2)	.405
Decreased Appetite	44 (23.8)	18 (26.1)	26 (22.4)	.921	Sweating	37 (20.0)	11 (15.9)	26 (22.4)	.637
Dry Mouth	43 (23.2)	10 (14.5)	33 (28.4)	.154	Dry Mouth	36 (19.5)	8 (11.6)	28 (24.1)	.195
Decreased Sleep	39 (21.1)	14 (20.3)	25 (21.6)	.507	Decreased Sleep	35 (18.9)	10 (14.5)	25 (21.6)	.873

Abbreviation: AD, anxious depression.

* Adjusted for multiple testing using the false discovery rate method (10 comparisons).

### 3.3 Relation between pretreatment anxious depression and number of side effects

According to the results of the covariate‐adjusted negative binomial regression, the number of side effects participants “experienced” and “had trouble” within the first interval was significantly higher among those with pretreatment anxious depression (*p* < .05; Table 3). In the second interval (weeks 2–4), participants with anxious depression did not have any significant effect on the number of side effects participants experienced or had trouble with (*p* > .05). All models met the goodness‐of‐fit chi‐square test (*p* < .05).

**TABLE 3:** Negative binomial and logistic regression results for pretreatment anxiety as a predictor of side effects

	Baseline to week 2			Weeks 2–4		
Negative binomial regression results^a^	Anxious depression—incidence rate ratio	95% CI	*p*	Anxious depression—incidence rate ratio	95% CI	*p*
Outcomes:						
Frequency—number of side effects experienced	1.38	1.08–1.75	.010	1.18	0.92–1.51	.202
Severity—number of side effects had trouble with	1.34	1.05–1.73	.026	1.17	0.89–1.52	.272

Logistic regression results^b^	Anxious depression—odds ratio	95% CI	*p*	Anxious depression—odds ratio	95% CI	*p*
Outcomes:						
Nervousness						
Frequency—had symptoms	3.03	1.42–6.87	.005	3.88	1.77–9.16	.001
Severity—had trouble	3.01	1.35–7.29	.010	4.57	1.94–12.19	.001
Agitation						
Frequency—had symptoms	1.99	0.91–4.54	.091	2.69	1.18–6.61	.023
Severity—had trouble	1.90	0.85–4.50	.127	3.16	1.31–8.57	.015

^a^ Negative binomial regression: number of side effects = pretreatment anxious depression + baseline depression severity + age + sex.

^b^ Logistic regression: presence of symptoms/trouble = pretreatment anxious depression + baseline depression severity + age + sex.

### 3.4 Relation between pretreatment anxious depression and specific side effects

Following significant associations identified by chi‐square and Fisher's exact tests for both 2‐week intervals (Table 2), covariate‐adjusted logistic regression analyses were further conducted on agitation and nervousness. Results showed that the odds of experiencing and having trouble with nervousness were significantly higher among participants with anxious depression throughout the whole treatment period from baseline to week 4 (baseline to week 2, *p* < .05; week 2–4, *p* < .05). A similar relationship was observed for agitation between weeks 2 and 4 only (*p* < .05; Table 3).

### 3.5 Escitalopram side effects: weeks 8–12

At week 8, all responders to escitalopram (*n* = 85) remained on monotherapy. Eighty‐three participants reported anxiety levels with 8 (9.6%) experiencing anxious depression. In the third (weeks 8–10) and fourth (weeks 10–12) 2‐week intervals, the top five most frequent and severe side effects largely overlapped with each other. Again, these side effects were related to CNS and sexual functioning: drowsiness, sweating, weakness/fatigue, dry mouth, and decreased libido (Table S1).

Participants with and without anxious depression at week 8 only differed significantly in the number of symptoms they had experienced between weeks 8 and 10 (median: 9.0 vs. 3.0; *W* = 407.5, *p* = .04). On the other hand, those with anxious depression at week 8 were significantly more likely to experience nervousness and agitation between weeks 8 and 10 (*p* < .05; Table S6) and to have trouble with nervousness between weeks 10 and 12 (*p* = .023; Tables S1 and S7).

### 3.6 Aripiprazole adjunctive therapy side effects: weeks 8–12

At week 8, all nonresponders to escitalopram (*n* = 92) switched to aripiprazole adjunctive therapy. Ninety participants reported anxiety symptoms, with 33 (36.7%) experiencing anxious depression. Between weeks 8 and 10, 89 participants reported receiving 2 mg of adjunctive aripiprazole. Between weeks 10 and 12, 45 and 42 participants reported receiving 2 mg and 4/5 mg of adjunctive aripiprazole, respectively. Between weeks 8 and 12, four out of the top five most frequent and severe side effects largely overlapped with each other and were related to CNS: drowsiness, decreased sleep, nervousness, and weakness fatigue (Table 4). Unlike the previous group, decreased libido was not present among the five most frequent or severe side effects in participants who switched to aripiprazole adjunctive therapy (Tables S4 and S5).

**TABLE 4:** Top 10 most frequent and severe aripiprazole side effects at between weeks 8 and 12

Weeks 8–10 frequency – have symptoms *n* (%)	Overall (*n* = 87)	No AD (*n* = 57)	AD (*n* = 30)	*p* ^a^	Weeks 8–10 severity—have trouble *n* (%)	Overall (*n* = 87)	No AD (*n* = 57)	AD (*n* = 30)	*p* ^a^
Drowsiness	34 (39.1)	21 (36.8)	13 (43.3)	1.000	Drowsiness	30 (34.5)	18 (31.6)	12 (40.0)	.984
Decreased sleep	33 (37.9)	23 (40.4)	10 (33.3)	1.000	Decreased sleep	30 (34.5)	21 (36.8)	9 (30.0)	.984
Agitation	26 (29.9)	12 (21.1)	14 (46.7)	.255	Weakness fatigue	23 (26.4)	13 (22.8)	10 (33.3)	.984
Nervousness	26 (29.9)	14 (24.6)	12 (40.0)	1.000	Nervousness	22 (25.3)	12 (21.1)	10 (33.3)	.984
Sweating	25 (28.7)	16 (28.1)	9 (30.0)	1.000	Dry mouth	19 (21.8)	12 (21.1)	7 (23.3)	1.000
Dry mouth	24 (27.6)	15 (26.3)	9 (30.0)	1.000	Agitation	19 (21.8)	7 (12.3)	12 (40.0)	.069
Weakness fatigue	24 (27.6)	14 (24.6)	10 (33.3)	1.000	Headache	19 (21.8)	11 (19.3)	8 (26.7)	.984
Weight gain	23 (26.4)	14 (24.6)	9 (30.0)	1.000	Sweating	18 (20.7)	11 (19.3)	7 (23.3)	1.000
Headache	21 (24.1)	13 (22.8)	8 (26.7)	1.000	Diarrhea	17 (19.5)	11 (19.3)	6 (20.0)	1.000
Diarrhea	19 (21.8)	13 (22.8)	6 (20.0)	1.000	Weight gain	16 (18.4)	9 (15.8)	7 (23.3)	.984

Weeks 10–12 frequency—have symptoms *n* (%)	Overall (*n* = 84)	No AD (*n* = 55)	AD (*n* = 29)	*p* ^a^	Weeks 10–12 severity—have trouble *n* (%)	Overall (*n* = 84)	No AD (*n* = 55)	AD (*n* = 29)	*p* ^a^
Agitation	34 (40.5)	19 (34.5)	15 (51.7)	1.000	Agitation	29 (34.5)	15 (27.3)	14 (48.3)	.923
Drowsiness	33 (39.3)	20 (36.4)	13 (44.8)	1.000	Drowsiness	28 (33.3)	17 (30.9)	11 (37.9)	.979
Decreased sleep	29 (34.5)	18 (32.7)	11 (37.9)	1.000	Decreased sleep	26 (31.0)	16 (29.1)	10 (34.5)	.994
Nervousness	27 (32.1)	16 (29.1)	11 (37.9)	1.000	Weakness fatigue	25 (29.8)	15 (27.3)	10 (34.5)	.979
Weakness fatigue	26 (31.0)	16 (29.1)	10 (34.5)	1.000	Nervousness	22 (26.2)	13 (23.6)	9 (31.0)	.979
Dry mouth	25 (29.8)	14 (25.5)	11 (37.9)	1.000	Dry mouth	20 (23.8)	11 (20.0)	9 (31.0)	.979
Weight gain	23 (27.4)	16 (29.1)	7 (24.1)	1.000	Postural hypotension	19 (22.6)	12 (21.8)	7 (24.1)	1.000
Sweating	21 (25.0)	14 (25.5)	7 (24.1)	1.000	Decreased libido	18 (21.4)	10 (18.2)	8 (27.6)	.979
Postural hypotension	21 (25.0)	13 (23.6)	8 (27.6)	1.000	Weight gain	18 (21.4)	14 (25.5)	4 (13.8)	.979
Decreased libido	20 (23.8)	11 (20.0)	9 (31.0)	1.000	Sweating	17 (20.2)	11 (20.0)	6 (20.7)	1.000

^a^ Adjusted for multiple testing using the False Discovery Rate method (10 comparisons).

For both 2‐week intervals, participants with and without anxious depression at week 8 did not differ in the number of symptoms they experienced or had trouble with (*p* > .05). In addition, there was no significant association between anxious depression and any specific side effects (*p* > .05; Table 4).

## 4 DISCUSSION

In this report, we examined the frequency and severity of side effects among MDD outpatient participants who received escitalopram in the first 8 weeks, followed by a further examination of those who remained on escitalopram (responders) or received aripiprazole adjunctive therapy in the second 8 weeks. For both therapies, we further examined whether pretreatment anxious depression was associated with the number and presence of specific side effects. There were four main findings: (1) side effects most frequently experienced by patients were also ones they had most trouble with; (2) these side effects for both therapies were mostly related to the CNS (i.e., drowsiness, nervousness, etc.); (3) pretreatment anxious depression was associated with higher number of side effects for escitalopram therapy only; and (4) pretreatment anxious depression was significantly associated with higher odds of experiencing and having trouble with anxiety‐related side effects (nervousness and agitation) for escitalopram only.

For escitalopram, we found that drowsiness, headache, weakness/fatigue, and nervousness were consistently among the top five most frequent and severe side effects between baseline to week 4, with 24–45% of participants reporting they had experienced and had trouble with them in the first and second 2‐week intervals since starting the treatment. Concomitantly, nausea was frequent or severe for 36.7–40.4% of participants between baseline and week 2, but decreased to 14.7–19.8% between weeks 2 and 4 (Tables S2 and S3). Exploratory results from responders to escitalopram use post‐week 8 showed that drowsiness, weakness/fatigue, and decreased libido remained among the top five most frequent and severe side effects between weeks 8 and 12, whereas nervousness and headache were among the top 10 side effects. The proportion of participants who experienced or had trouble with side effects were between 20 and 36% after the week 8 of therapy, thus indicating improvement. Clinically, findings suggest that drowsiness, weakness fatigue, decreased libido, and, to a lesser extent, nervousness and headache persist as side effects over the course of 16‐week escitalopram therapy despite associated improvements in depression symptoms, whereas nausea appears as a major side effect predominantly in the first four weeks of escitalopram therapy. As these side effects are commonly associated with early SSRI discontinuation (Baldwin et al., 2007; Bull et al., 2002; Kroenke et al., 2001), providing patient information, careful monitoring of and rapidly intervening in these potential adverse events are pivotal to maintaining treatment adherence, especially in the first weeks after treatment initiation (Bull et al., 2002).

The side effects for escitalopram, as reported in the current study, are in line with previous research. A pooled analysis (Baldwin et al., 2007) across 23 RCTs on escitalopram (14 in MDD; eight in anxiety disorders; and one in obsessive‐compulsive disorder) revealed that adverse events with the highest incidence rates (IRs) in the first 8 weeks were: headache (18%), nausea (17%), sexual dysfunction (10%; including decreased libido, delayed ejaculation, etc.), insomnia (8%), and fatigue (8%). Similarly, the current study also found nausea, headache, decreased libido, weakness fatigue to be among the most frequent and severe side effects between baseline and week 4. In addition, the pooled analysis also found that the point prevalence of nausea was initially higher for escitalopram than for placebo, but eventually decreased to a similar to placebo level after 30 days (Baldwin et al., 2007). Likewise, our findings showed that the proportion of escitalopram recipients who experienced nausea declined from 40% in the first 2‐weeks to 20% in the second 2‐weeks and to 13% between weeks 10 and 12. Together, our findings suggest that certain gastrointestinal side effects are more transient compared with the CNS and sexual functioning side effects among escitalopram recipients.

However, the present study differed from the pooled analysis in two ways. First, the proportion of participants who experienced and had trouble with side effects was higher than the incidence and prevalence rates reported in the pooled analysis. Second, drowsiness consistently ranked as the most frequent and severe side effect in the present study, whereas it did not in the pooled analysis. These differences may be due to varying methods in side‐effect assessment. TSES uses specific side‐effect questioning rather than spontaneous reporting and investigator observation used in studies in the pooled analysis (Baldwin et al., 2007). Although this form of questioning assesses side effects in a more standardized and comprehensive manner, it may also introduce suggestibility and increase side‐effect reporting. Alternatively, spontaneous reporting might also have caused underreporting of mild or transient side effects in the pooled analysis. Additionally, the inclusion of patients with other disorders in the pooled analysis might be responsible for different rates of adverse events from this study. Finally, escitalopram mean daily dose was not considered when comparing these studies, as it may influence the side effect profile (Polychroniou et al., 2018).

For week 8, among nonresponders who switched to aripiprazole adjunctive therapy, drowsiness, nervousness, and decreased sleep were among both the top five most frequent and severe effects between weeks 8 and 10, with 21–38% of participants reporting they had experienced or had trouble with them. Between weeks 10 and 12, drowsiness, nervousness, and decreased sleep still persisted, whereas agitation and weakness fatigue emerged as a top five most frequent and severe side effects. There were 26–40% of participants who had experienced or had trouble with them, suggesting a lack of improvement in side effects. Clinically, findings indicate that side effects related to CNS activation persist among patients who switched to adjunctive therapy.

Findings from the present study on aripiprazole adjunctive therapy were mostly in line with previous research. Two RCT studies included an initial 8‐week period, where MDD patients received an antidepressant of clinician's choice plus single‐blind adjunctive placebo, and those who did not respond were randomized to either adjunctive placebo or aripiprazole adjunctive therapy (Berman et al., 2009; Marcus et al., 2008). Results showed that akathisia was the most frequent side effect with an IR of ≥15%. Besides akathisia, other side effects that had an IR between 5 and 10% and twice that of placebo were fatigue, somnolence, restlessness, insomnia, and tremor. These latter side effects were similar to the sleep and anxiety‐related side effects found in the present study. Conversely, akathisia was not reported as a top side effect in the present study potentially because TSES does not include it as an item. Furthermore, unlike the two RCTs, the present study did not utilize akathisia‐specific scales such as the Barnes Akathisia Clinical Assessment. That being said, agitation and nervousness, which shares similar features with akathisia, were reported as the top side effects and hence may suggest the presence of akathisia. Additionally, this study only administered the TSES for patients on adjunctive aripiprazole for a 4‐week period (weeks 8–12), and this represents an important limitation to our study. Akathisia, the most common side effect of aripiprazole therapy (Casey & Canal, 2017) is associated with higher doses of this antipsychotic drug (Lenze et al., 2015) with an RCT reporting the median dose of 7 mg for its onset (Lenze et al., 2015). However, literature suggests that other aripiprazole side effects, such as weight gain (Simon et al., 2009) and other metabolic adverse events (e.g., body fat, total cholesterol, triglycerides, glucose, or insulin concentrations), are either dose independent or not different from placebo respectively (Lenze et al., 2015; Simon et al., 2009). With the growing use of atypical agents including aripiprazole, which frequently induces akathisia (Cheon et al., 2017; Poyurovsky, 2010; Thomas et al., 2015), additional items should be incorporated into the TSES.

On the other hand, unlike responders to escitalopram monotherapy, the adjunctive therapy group did not have decreased libido present among the top 5 most significant side effects, which suggests that aripiprazole augmentation may have improved libido. These results are in line with previous studies (Cheon et al., 2017; Fava et al., 2011), which show that adjunctive aripiprazole significantly reduced sexual dysfunction in patients with MDD on antidepressant use and may be possibly due to its partial dopamine D2 and serotonin 5HT1A receptor agonism and partial serotonin 5HT2A receptor antagonism (Kozian, 2020; Mété et al., 2016; Montejo et al., 2019; Stahl, 2013).

In the present study, pretreatment anxious depression was significantly associated with a greater number of side effects as well as experiencing/having trouble with specific side effects related to anxiety (nervousness and agitation) among escitalopram recipients between baseline to week 4 and weeks 8 and 12. We hypothesize that patients with anxious depression might be more sensitive to antidepressant activating effects in the initial weeks of treatment. These findings were in line with a STAR*D study, which showed that anxious depression is associated with greater overall frequency and intensity/severity of side effects among citalopram recipients (Fava et al., 2008). However, our findings differed from previous escitalopram studies that found the mean number of overall side effects and the frequency of specific side effects to not significantly differ between those with and without anxious depression (Boulenger et al., 2010; Polychroniou et al., 2018). These discrepancies could be due to previous escitalopram studies using spontaneous reporting, which has been associated with underreporting of side effects (Hazell & Shakir, 2006). It could also be due to previous studies defining anxious depression with HAM‐A, whereas the present study defined with GAD‐7. The former is a 14‐item clinician‐based scale that comprises both psychological and somatic symptoms (Hamilton, 1959), whereas the latter is a 7‐item self‐reported questionnaire that primarily focuses on psychological symptoms of anxiety (Spitzer et al., 2006). Future studies could continue to examine these associations using standardized side effect scales and consistent definitions of anxious depression to produce replicable findings (Ionescu et al., 2013).

On the other hand, associations between anxious depression and side effects were not observed with aripiprazole adjunctive therapy in the present report. These findings were in line with a previous aripiprazole study that used spontaneous reporting and found the side effect profile to not differ significantly between those with and without anxious depression (Trivedi et al., 2008). Additionally, there is evidence that antidepressants have distinct pharmacological and side effect profiles (Anderson et al., 2012); for example, sertraline is associated with a higher incidence of diarrhea (Cipriani et al., 2009), and paroxetine with greater sedation, constipation, and sexual dysfunction than other SSRIs (Marks et al., 2008). Together, findings suggest that the association between anxious depression and number of side effects could depend on the antidepressant therapy used. Patients who receive escitalopram and have anxious depression may require closer monitoring of side effects in the initial weeks of treatment commencement, particularly the most frequent and severe ones (e.g., drowsiness, nausea, nervousness). Future studies could continue to examine this association using data from comparative clinical trials.

Clinically, having upward to 45% of participants experiencing or having with trouble with side effects, along with how anxious depression is associated with more side effects, points toward the importance of routinely utilizing standardized side effect scales during antidepressant therapy. Scales like TSES allow greater sensitivity to capturing patient reported adverse effects and has been reported to pick up 20 times more than alternative patient‐administered questionnaires (Zimmerman et al., 2010). This provides a promising opportunity for physicians to better anticipate side effects and to implement active physician–patient communication regarding the side effect profiles of escitalopram and aripiprazole adjunctive therapy to ensure patient compliance and adherence (Hu et al., 2004).

### 4.1 Limitations

This study has several limitations. First, a placebo control group was not included in the trial, so we are unable to compare side effects across the placebo and treatment groups. Second, the sample size was small between weeks 8 and 12 for the aripiprazole adjunctive and escitalopram therapy cohorts. Therefore, we were unable to conduct covariate‐adjusted logistic and negative binomial regression models. Third, the side effects in our sample are specific for adults and further investigation would be needed to examine other populations (e.g., geriatric). Fourth, there was a significantly higher proportion of female participants in the sample without side effects data. Although the lack of data was not due to adverse events, our analysis sample may be affected by potential selection biases.

## 5 CONCLUSION

In summary, the present study demonstrates the utility of a standardized side effects questionnaire (TSES) and extends previously reported adverse effects data in CAN‐BIND‐1 (Kennedy et al., 2019). For both escitalopram and aripiprazole adjunctive therapies, side effects related to the CNS and, to a lesser extent, sexual functioning are the most frequent and troubling. Having pretreatment anxious depression is associated with significantly higher number of side effects during the initial 2‐weeks of escitalopram therapy. It also provides predictive utility to the presence of anxiety‐related side effects. Together, findings suggest that pretreatment anxious depression is a factor that predisposes MDD patients to experiencing more side effects. Therefore, for those with anxious depression, physician–patient communication regarding anticipated side effects may be particularly important to potentially improve antidepressant compliance.

## FUNDING

This project did not receive any funding support. The data used for this project were collected as part of the Canadian Biomarker Integration Network in Depression (CAN‐BIND) program. CAN‐BIND is an Integrated Discovery Program supported by the Ontario Brain Institute, which is an independent non‐profit corporation funded partially by the Ontario Government. Additional funding was provided to CAN‐BIND by the Canadian Institutes of Health Research (CIHR), Lundbeck, Bristol‐Myers Squibb, and Servier.

CWE, YK, RP, ID, KH, and WL declared no potential conflicts of interest with respect to the research, authorship, and/or publication of this article. BF has received grant/research support from Alternative Funding Plan Innovations Award, Brain and Behavior Research Foundation, Canadian Institutes of Health Research (CIHR), Hamilton Health Sciences Foundation, J.P. Bickell Foundation, Ontario Brain Institute (OBI), Ontario Mental Health Foundation (OMHF), Society for Women's Health Research, Teresa Cascioli Charitable Foundation, Eli Lilly and Pfizer, and has received consultant and/or speaker fees from AstraZeneca, Bristol‐Myers Squibb, Canadian Psychiatric Association, CANMAT, Daiichi Sankyo, Lundbeck, Pfizer, Servier and Sunovion. RVM has received consulting and speaking honoraria from AbbVie, Allergan, Janssen, KYE, Lundbeck, Otsuka, and Sunovion, and research grants from CAN‐BIND, CIHR, Janssen, Lallemand, Lundbeck, Nubiyota, OBI and OMHF. AVR has received speaker and consultant honoraria or research funds from Bristol Myers Squibb, Canadian Depression Research and Intervention Network, Canadian Foundation for Innovation and the Ministry of Economic Development and Innovation, Canadian Institutes of Health Research, Grand Challenges Canada, Janssen, Lundbeck, Ontario Mental Health Foundation, Pfizer and Sunovion. SVP has been a consultant to Takeda, Bristol Myers Squibb, Lundbeck; has had a research contract with Assurex; has equity in Mensante. SR holds a patent ‘Teneurin C‐Terminal Associated Peptides (TCAP) and methods and uses thereof.’ Inventors: David Lovejoy, R.B. Chewpoy, Dalia Barsyte, Susan Rotzinger. RWL has received speaker and consultant honoraria or research funds from AstraZeneca, Brain Canada, Bristol‐Myers Squibb, the Canadian Institutes of Health Research (CIHR), the Canadian Network for Mood and Anxiety Treatments, the Canadian Psychiatric Association, Eli Lilly, Janssen, Lundbeck, Lundbeck Institute, Medscape, Otsuka, Pfizer, Servier, St. Jude Medical, Takeda, the University Health Network Foundation, and Vancouver Coastal Health Research Institute. SHK has received honoraria or research funds from Abbott, Alkermes, Allergan, Boehringer Ingelheim, Brain Canada, CIHR, Janssen, Lundbeck, Lundbeck Institute, Ontario Brain Institute, Ontario Research Fund, Otsuka, Pfizer, Servier, Sunovion, Sun Pharmaceuticals, and holds stock in Field Trip Health. VB is supported by an Academic Scholar Award from the University of Toronto's Department of Psychiatry and has received research support from CIHR, Brain & Behavior Foundation, MOH Innovation Funds, RCPSC, Department of Defense, Canada, and an investigator‐initiated trial from Roche Canada.

### TRANSPARENT PEER REVIEW

The peer review history for this article is available at https://publons.com/publon/10.1002/brb3.2555

## Supporting information

Supporting Information

## Data Availability

Data are not publicly available in accordance with ethics approval given by the ethics board from the participating university. Interested investigators may submit inquiries to the corresponding author.
