# Supplementary material for: Pretreatment anxious depression as a predictor of side effect frequency and severity in escitalopram and aripiprazole adjunctive therapy
Source: Brain Behav. 2022 Mar 25;12(5):e2555. doi: 10.1002/brb3.2555 (PMC9120722; doi:10.1002/brb3.2555)
Supplement: Supplementary file 1 — Supporting Information [file BRB3-12-e2555-s001.docx]

**Appendices**

| **Table 1.** Top Ten Most Frequent and Severe Escitalopram Side Effects at Between Weeks 8 to 12 | | | | | | | | | |
| --- | --- | --- | --- | --- | --- | --- | --- | --- | --- |
| **Weeks 8 to 10 Frequency –**  **Have Symptoms**  **n (%)** | **Overall**  **(n = 79)** | **No AD**  **(n = 71)** | **AD**  **(n = 8)** | ***p*^†^** | **Weeks 8 to 10 Severity –**  **Have Trouble**  **n (%)** | **Overall**  **(n = 79)** | **No AD**  **(n = 71)** | **AD**  **(n = 8)** | ***p*^†^** |
| Drowsiness | 24 (30.4) | 22 (31.0) | 2 (25.0) | 1.000 | Drowsiness | 21 (26.6) | 20 (28.2) | 1 (12.5) | .674 |
| Sweating | 20 (25.3) | 17 (23.9) | 3 (37.5) | .513 | Dry Mouth | 16 (20.3) | 13 (18.3) | 3 (37.5) | .435 |
| Weakness Fatigue | 18 (22.8) | 15 (21.1) | 3 (37.5) | .513 | Weakness Fatigue | 16 (20.3) | 13 (18.3) | 3 (37.5) | .435 |
| Dry Mouth | 16 (20.3) | 13 (18.3) | 3 (37.5) | .513 | Decreased Libido | 15 (19.0) | 12 (16.9) | 3 (37.5) | .343 |
| Decreased Libido | 16 (20.3) | 13 (18.3) | 3 (37.5) | .513 | Sweating | 15 (19.0) | 12 (16.9) | 3 (37.5) | .342 |
| **Agitation** | **15 (19.0)** | **10 (14.1)** | **5 (62.5)** | **.026** | Agitation | 12 (15.2) | 9 (12.7) | 3 (37.5) | .325 |
| **Nervousness** | **14 (17.7)** | **9 (12.7)** | **5 (62.5)** | **.026** | Nervousness | 12 (15.2) | 8 (11.3) | 4 (50.0) | .161 |
| Anorgasmia | 13 (16.5) | 13 (18.3) | 0 (0.0) | .513 | Anorgasmia | 11 (13.9) | 11 (15.5) | 0 (0.0) | .658 |
| Decreased Appetite | 13 (16.5) | 10 (14.1) | 3 (37.5) | .400 | Decreased Sleep | 11 (13.9) | 9 (12.7) | 2 (25.0) | .435 |
| Postural Hypotension | 13 (16.5) | 11 (15.5) | 2 (25.0) | .680 | Weight Gain | 11 (13.9) | 8 (11.3) | 3 (37.5) | .325 |
|  |  |  |  |  |  |  |  |  |  |
| **Weeks 10 to 12 Frequency –**  **Have Symptoms**  **n (%)** | **Overall**  **(n = 80)** | **No AD**  **(n = 72)** | **AD**  **(n = 8)** | ***p*^†^** | **Weeks 10 to 12 Severity – Have Trouble**  **n (%)** | **Overall**  **(n = 80)** | **No AD**  **(n = 72)** | **AD**  **(n = 8)** | ***p*^†^** |
| Drowsiness | 29 (36.2) | 26 (36.1) | 3 (37.5) | 1.000 | Drowsiness | 27 (33.8) | 24 (33.3) | 3 (37.5) | 1.000 |
| Sweating | 18 (22.5) | 15 (20.8) | 3 (37.5) | .463 | Sweating | 16 (20.0) | 13 (18.1) | 3 (37.5) | .277 |
| Nervousness | 17 (21.2) | 12 (16.7) | 5 (62.5) | .094 | Weakness Fatigue | 16 (20.0) | 12 (16.7) | 4 (50.0) | .117 |
| Weakness Fatigue | 17 (21.2) | 13 (18.1) | 4 (50.0) | .146 | Dry Mouth | 15 (18.8) | 13 (18.1) | 2 (25.0) | .711 |
| Decreased Libido | 16 (20.0) | 13 (18.1) | 3 (37.5) | .323 | Decreased Libido | 14 (17.5) | 11 (15.3) | 3 (37.5) | .234 |
| Dry Mouth | 15 (18.8) | 13 (18.1) | 2 (25.0) | .711 | Anorgasmia | 13 (16.2) | 13 (18.1) | 0 (0.0) | .425 |
| Decreased Sleep | 15 (18.8) | 13 (18.1) | 2 (25.0) | .323 | Decreased Sleep | 13 (16.2) | 11 (15.3) | 2 (25.0) | .234 |
| Agitation | 14 (17.5) | 10 (13.9) | 4 (50.0) | .094 | Headache | 13 (16.2) | 9 (12.5) | 4 (50.0) | .071 |
| Headache | 14 (17.5) | 10 (13.9) | 4 (50.0) | .094 | **Nervousness** | **13 (16.2)** | **8 (11.1)** | **5 (62.5)** | **.023** |
| Anorgasmia | 13 (16.2) | 13 (18.1) | 0 (0.0) | .46 | Agitation | 12 (15.0) | 8 (11.1) | 4 (50.0) | .071 |

Abbreviations: AD = Anxious Depression

^†^adjusted for multiple testing using the False Discovery Rate method; Statistical significance for all tests were set at p < .05.

When cell count was five and higher, Chi-square test was conducted

When cell count was lower than 5, Fisher’s test was conducted

| **Table 2**. Summary of Frequency and Severity of Side Effects Between Baseline to 2 (n = 188) | | | | | | | | | |  |
| --- | --- | --- | --- | --- | --- | --- | --- | --- | --- | --- |
| **Baseline to week 2 Frequency – Have Symptoms**  **n (%)** | **Missing** | **Overall**  **(n = 188)** | **No AD**  **(n = 69)** | **AD**  **(n = 119)** | **Baseline to week 2 Severity – Have Trouble**  **n (%)** | **Missing** | **Overall**  **(n = 188)** | **No AD**  **(n = 69)** | **AD**  **(n = 119)** | |
| Drowsiness | 1 (0.5) | 85 (45.2) | 26 (37.7) | 59 (49.6) | Drowsiness | 1 (0.5) | 73 (38.8) | 22 (31.9) | 51 (42.9) | |
| Nausea | 0 (0) | 76 (40.4) | 27 (39.1) | 49 (41.2) | Nausea | 0 (0) | 69 (36.7) | 24 (34.8) | 45 (37.8) | |
| Headache | 0 (0) | 67 (35.6) | 21 (30.4) | 46 (38.7) | Headache | 0 (0) | 58 (30.9) | 17 (24.6) | 41 (34.5) | |
| Weakness Fatigue | 0 (0) | 60 (31.9) | 19 (27.5) | 41 (34.5) | Weakness Fatigue | 0 (0) | 53 (28.2) | 18 (26.1) | 35 (29.4) | |
| Nervousness | 0 (0) | 56 (29.8) | 11 (15.9) | 45 (37.8) | Nervousness | 0 (0) | 49 (26.1) | 9 (13.0) | 40 (33.6) | |
| Dyspepsia | 0 (0) | 51 (27.1) | 19 (27.5) | 32 (26.9) | Agitation | 0 (0) | 47 (25.0) | 10 (14.5) | 37 (31.1) | |
| Agitation | 0 (0) | 51 (27.1) | 11 (15.9) | 40 (33.6) | Dyspepsia | 0 (0) | 46 (24.5) | 17 (24.6) | 29 (24.4) | |
| Dry Mouth | 0 (0) | 47 (25.0) | 12 (17.4) | 35 (29.4) | Dry Mouth | 0 (0) | 41 (21.8) | 10 (14.5) | 31 (26.1) | |
| Decreased Appetite | 0 (0) | 45 (23.9) | 16 (23.2) | 29 (24.4) | Decreased Sleep | 0 (0) | 37 (19.7) | 12 (17.4) | 25 (21.0) | |
| Increased Sleep | 0 (0) | 42 (22.3) | 14 (20.3) | 28 (23.5) | Decreased Appetite | 0 (0) | 33 (17.6) | 11 (15.9) | 22 (18.5) | |
| Decreased Sleep | 0 (0) | 41 (21.8) | 15 (21.7) | 26 (21.8) | Increased Sleep | 0 (0) | 33 (17.6) | 12 (17.4) | 21 (17.6) | |
| Diarrhea | 0 (0) | 37 (19.7) | 9 (13.0) | 28 (23.5) | Sweating | 0 (0) | 32 (17.0) | 7 (10.1) | 25 (21.0) | |
| Sweating | 0 (0) | 36 (19.1) | 9 (13.0) | 27 (22.7) | Dizziness | 0 (0) | 31 (16.5) | 10 (14.5) | 21 (17.6) | |
| Decreased Libido | 1 (0.5) | 33 (17.6) | 8 (11.6) | 25 (21.0) | Decreased Libido | 1 (0.5) | 31 (16.5) | 7 (10.1) | 24 (20.2) | |
| Dizziness | 0 (0) | 32 (17.0) | 10 (14.5) | 22 (18.5) | Diarrhea | 0 (0) | 30 (16.0) | 5 (7.2) | 25 (21.0) | |
| Increased Appetite | 0 (0) | 30 (16.0) | 7 (10.1) | 23 (19.3) | Abdominal Pain | 0 (0) | 27 (14.4) | 7 (10.1) | 20 (16.8) | |
| Abdominal Pain | 0 (0) | 28 (14.9) | 8 (11.6) | 20 (16.8) | Anorgasmia | 5 (2.7) | 23 (12.2) | 7 (10.1) | 16 (13.4) | |
| Anorgasmia | 5 (2.7) | 23 (12.2) | 7 (10.1) | 16 (13.4) | Increased Appetite | 0 (0) | 23 (12.2) | 6 (8.7) | 17 (14.3) | |
| Tremor | 0 (0) | 23 (12.2) | 9 (13.0) | 14 (11.8) | Constipation | 1 (0.5) | 20 (10.6) | 5 (7.2) | 15 (12.6) | |
| Postural Hypotension | 0 (0) | 23 (12.2) | 9 (13.0) | 14 (11.8) | Tremor | 0 (0) | 20 (10.6) | 8 (11.6) | 12 (10.1) | |
| Constipation | 1 (0.5) | 22 (11.7) | 4 (5.8) | 18 (15.1) | Postural Hypotension | 0 (0) | 19 (10.1) | 9 (13.0) | 10 (8.4) | |
| Flushing | 0 (0) | 21 (11.2) | 4 (5.8) | 17 (14.3) | Flushing | 0 (0) | 17 (9.0) | 4 (5.8) | 13 (10.9) | |
| Blurred Vision | 0 (0) | 19 (10.1) | 4 (5.8) | 15 (12.6) | Blurred Vision | 0 (0) | 15 (8.0) | 3 (4.3) | 12 (10.1) | |
| Weight Gain | 1 (0.5) | 19 (10.1) | 4 (5.8) | 15 (12.6) | Delayed Ejaculation | 116 (61.7) | 11 (5.9) | 3 (4.3) | 8 (6.7) | |
| Increased Libido | 2 (1.1) | 14 (7.4) | 3 (4.3) | 11 (9.2) | Muscle Contraction | 0 (0) | 9 (4.8) | 0 (0.0) | 9 (7.6) | |
| Weight Loss | 0 (0) | 14 (7.4) | 2 (2.9) | 12 (10.1) | Weight Gain | 1 (0.5) | 9 (4.8) | 2 (2.9) | 7 (5.9) | |
| Delayed Ejaculation | 116 (61.7) | 14 (7.4) | 4 (5.8) | 10 (8.4) | Weight Loss | 0 (0) | 5 (2.7) | 1 (1.4) | 4 (3.4) | |
| Muscle Contraction | 0 (0) | 13 (6.9) | 0 (0.0) | 13 (10.9) | Edema | 0 (0) | 4 (2.1) | 1 (1.4) | 3 (2.5) | |
| Edema | 0 (0) | 6 (3.2) | 3 (4.3) | 3 (2.5) | Erectile Dysfunction | 116 (61.7) | 4 (2.1) | 2 (2.9) | 2 (1.7) | |
| Erectile Dysfunction | 116 (61.7) | 5 (2.7) | 2 (2.9) | 3 (2.5) | Increased Libido | 2 (1.1) | 2 (1.1) | 0 (0.0) | 2 (1.7) | |
| Premature Ejaculation | 116 (61.7) | 1 (0.5) | 0 (0.0) | 1 (0.8) | Premature Ejaculation | 116 (61.7) | 0 (0.0) | 0 (0.0) | 0 (0.0) | |

Abbreviations: AD = Anxious Depression

| **Table 3**. Summary of Frequency and Severity of Side Effects Between Weeks 2 to 4 (n = 185) | | | | | | | | | |  |
| --- | --- | --- | --- | --- | --- | --- | --- | --- | --- | --- |
| **Weeks 2 to 4 Frequency –**  **Have Symptoms**  **n (%)** | **Missing** | **Overall**  **(n = 185)** | **No AD**  **(n = 69)** | **AD**  **(n = 116)** | **Weeks 2 to 4 Severity – Have Trouble**  **n (%)** | **Missing** | **Overall**  **(n = 185)** | **No AD**  **(n = 69)** | **AD**  **(n = 116)** | |
| Drowsiness | 0 (0) | 80 (43.2) | 29 (42.0) | 51 (44.0) | Drowsiness | 0 (0) | 71 (38.4) | 25 (36.2) | 46 (39.7) | |
| Weakness Fatigue | 0 (0) | 65 (35.1) | 24 (34.8) | 41 (35.3) | Weakness Fatigue | 0 (0) | 62 (33.5) | 24 (34.8) | 38 (32.8) | |
| Nervousness | 0 (0) | 54 (29.2) | 10 (14.5) | 44 (37.9) | Nervousness | 0 (0) | 47 (25.4) | 7 (10.1) | 40 (34.5) | |
| Decreased Libido | 1 (0.5) | 50 (27.0) | 17 (24.6) | 33 (28.4) | Headache | 0 (0) | 46 (24.9) | 13 (18.8) | 33 (28.4) | |
| Headache | 0 (0) | 49 (26.5) | 15 (21.7) | 34 (29.3) | Decreased Libido | 1 (0.5) | 44 (23.8) | 15 (21.7) | 29 (25.0) | |
| Increased Sleep | 0 (0) | 46 (24.9) | 14 (20.3) | 32 (27.6) | Agitation | 0 (0) | 40 (21.6) | 7 (10.1) | 33 (28.4) | |
| Agitation | 0 (0) | 45 (24.3) | 9 (13.0) | 36 (31.0) | Anorgasmia | 2 (1.1) | 38 (20.5) | 18 (26.1) | 20 (17.2) | |
| Decreased Appetite | 1 (0.5) | 44 (23.8) | 18 (26.1) | 26 (22.4) | Sweating | 0 (0) | 37 (20.0) | 11 (15.9) | 26 (22.4) | |
| Dry Mouth | 0 (0) | 43 (23.2) | 10 (14.5) | 33 (28.4) | Dry Mouth | 0 (0) | 36 (19.5) | 8 (11.6) | 28 (24.1) | |
| Sweating | 0 (0) | 43 (23.2) | 12 (17.4) | 31 (26.7) | Increased Sleep | 0 (0) | 36 (19.5) | 12 (17.4) | 24 (20.7) | |
| Anorgasmia | 1 (0.1) | 39 (21.1) | 18 (26.1) | 21 (18.1) | Decreased Appetite | 1 (0.5) | 35 (18.9) | 14 (20.3) | 21 (18.1) | |
| Decreased Sleep | 0 (0) | 39 (21.1) | 14 (20.3) | 25 (21.6) | Decreased Sleep | 0 (0) | 35 (18.9) | 10 (14.5) | 25 (21.6) | |
| Diarrhea | 0 (0) | 38 (20.5) | 12 (17.4) | 26 (22.4) | Diarrhea | 0 (0) | 33 (17.8) | 11 (15.9) | 22 (19.0) | |
| Nausea | 0 (0) | 37 (20.0) | 14 (20.3) | 23 (19.8) | Nausea | 0 (0) | 28 (15.1) | 11 (15.9) | 17 (14.7) | |
| Dyspepsia | 0 (0) | 30 (16.2) | 8 (11.6) | 22 (19.0) | Postural Hypotension | 0 (0) | 27 (14.6) | 11 (15.9) | 16 (13.8) | |
| Postural Hypotension | 0 (0) | 30 (16.2) | 11 (15.9) | 19 (16.4) | Constipation | 0 (0) | 25 (13.5) | 7 (10.1) | 18 (15.5) | |
| Constipation | 0 (0) | 27 (14.6) | 7 (10.1) | 20 (17.2) | Dyspepsia | 0 (0) | 24 (13.0) | 5 (7.2) | 19 (16.4) | |
| Delayed Ejaculation | 116 (62.7) | 27 (14.6) | 14 (20.3) | 13 (11.2) | Dizziness | 0 (0) | 23 (12.4) | 10 (14.5) | 13 (11.2) | |
| Dizziness | 0 (0) | 23 (12.4) | 10 (14.5) | 13 (11.2) | Delayed Ejaculation | 116 (62.7) | 23 (12.4) | 12 (17.4) | 11 (9.5) | |
| Tremor | 0 (0) | 23 (12.4) | 9 (13.0) | 14 (12.1) | Weight Gain | 3 (1.6) | 20 (10.8) | 6 (8.7) | 14 (12.1) | |
| Weight Gain | 3 (0.02) | 23 (12.4) | 8 (11.6) | 15 (12.9) | Tremor | 0 (0) | 19 (10.3) | 8 (11.6) | 11 (9.5) | |
| Increased Appetite | 0 (0) | 23 (12.4) | 9 (13.0) | 14 (12.1) | Increased Appetite | 0 (0) | 19 (10.3) | 8 (11.6) | 11 (9.5) | |
| Abdominal Pain | 0 (0) | 21 (11.4) | 4 (5.8) | 17 (14.7) | Abdominal Pain | 0 (0) | 17 (9.2) | 3 (4.3) | 14 (12.1) | |
| Muscle Contraction | 1 (0.5) | 21 (11.4) | 4 (5.8) | 17 (14.7) | Muscle Contraction | 1 (0.5) | 16 (8.6) | 3 (4.3) | 13 (11.2) | |
| Weight Loss | 1 (0.5) | 19 (10.3) | 6 (8.7) | 13 (11.2) | Blurred Vision | 0 (0) | 15 (8.1) | 6 (8.7) | 9 (7.8) | |
| Blurred Vision | 0 (0) | 17 (9.2) | 6 (8.7) | 11 (9.5) | Flushing | 0 (0) | 13 (7.0) | 3 (4.3) | 10 (8.6) | |
| Flushing | 0 (0) | 15 (8.1) | 3 (4.3) | 12 (10.3) | Weight Loss | 1 (0.5) | 9 (4.9) | 3 (4.3) | 6 (5.2) | |
| Increased Libido | 1 (0.5) | 13 (7.0) | 3 (4.3) | 10 (8.6) | Erectile Dysfunction | 116 (62.7) | 7 (3.8) | 3 (4.3) | 4 (3.4) | |
| Erectile Dysfunction | 116 (62.7) | 7 (3.8) | 3 (4.3) | 4 (3.4) | Edema | 0 (0) | 5 (2.7) | 1 (1.4) | 4 (3.4) | |
| Edema | 0 (0) | 4 (2.2) | 1 (1.4) | 3 (2.6) | Increased Libido | 1 (0.5) | 3 (1.6) | 1 (1.4) | 2 (1.7) | |
| Premature Ejaculation | 116 (62.7) | 0 (0.0) | 0 (0.0) | 0 (0.0) | Premature Ejaculation | 116 (62.7) | 0 (0.0) | 0 (0.0) | 0 (0.0) | |

Abbreviations: AD = Anxious Depression

**Table 4.** Summary of Weeks 8 to 10 Frequency and Severity of Side Effects in Aripiprazole Adjunctive Therapy Cohort (n = 87)^a^

| **Weeks 8 to 10 Frequency – Have Symptoms**  **n (%)** | **Overall**  **(n = 87)** | **No AD**  **(n = 57)** | **AD**  **(n = 30)** | **Weeks 8 to 10 Severity – Have Trouble**  **n (%)** | **Overall**  **(n = 87)** | **No AD**  **(n = 57)** | **AD**  **(n = 30)** |
| --- | --- | --- | --- | --- | --- | --- | --- |
| Drowsiness | 34 (39.1) | 21 (36.8) | 13 (43.3) | Drowsiness | 30 (34.5) | 18 (31.6) | 12 (40.0) |
| Decreased Sleep | 33 (37.9) | 23 (40.4) | 10 (33.3) | Decreased Sleep | 30 (34.5) | 21 (36.8) | 9 (30.0) |
| Agitation | 26 (29.9) | 12 (21.1) | 14 (46.7) | Weakness Fatigue | 23 (26.4) | 13 (22.8) | 10 (33.3) |
| Nervousness | 26 (29.9) | 14 (24.6) | 12 (40.0) | Nervousness | 22 (25.3) | 12 (21.1) | 10 (33.3) |
| Sweating | 25 (28.7) | 16 (28.1) | 9 (30.0) | Dry Mouth | 19 (21.8) | 12 (21.1) | 7 (23.3) |
| Dry Mouth | 24 (27.6) | 15 (26.3) | 9 (30.0) | Agitation | 19 (21.8) | 7 (12.3) | 12 (40.0) |
| Weakness Fatigue | 24 (27.6) | 14 (24.6) | 10 (33.3) | Headache | 19 (21.8) | 11 (19.3) | 8 (26.7) |
| Weight Gain | 23 (26.4) | 14 (24.6) | 9 (30.0) | Sweating | 18 (20.7) | 11 (19.3) | 7 (23.3) |
| Headache | 21 (24.1) | 13 (22.8) | 8 (26.7) | Diarrhea | 17 (19.5) | 11 (19.3) | 6 (20.0) |
| Diarrhea | 19 (21.8) | 13 (22.8) | 6 (20.0) | Weight Gain | 16 (18.4) | 9 (15.8) | 7 (23.3) |
| Increased Appetite | 18 (20.7) | 13 (22.8) | 5 (16.7) | Nausea | 16 (18.4) | 10 (17.5) | 6 (20.0) |
| Nausea | 17 (19.5) | 10 (17.5) | 7 (23.3) | Postural Hypotension | 15 (17.2) | 9 (15.8) | 6 (20.0) |
| Postural Hypotension | 17 (19.5) | 11 (19.3) | 6 (20.0) | Decreased Appetite | 14 (16.1) | 7 (12.3) | 7 (23.3) |
| Dyspepsia | 16 (18.4) | 12 (21.1) | 4 (13.3) | Dyspepsia | 13 (14.9) | 11 (19.3) | 2 (6.7) |
| Dizziness | 16 (18.4) | 9 (15.8) | 7 (23.3) | Increased Appetite | 13 (14.9) | 9 (15.8) | 4 (13.3) |
| Decreased Appetite | 16 (18.4) | 9 (15.8) | 7 (23.3) | Dizziness | 12 (13.8) | 6 (10.5) | 6 (20.0) |
| Decreased Libido | 15 (17.2) | 7 (12.3) | 8 (26.7) | Decreased Libido | 12 (13.8) | 7 (12.3) | 5 (16.7) |
| Increased Sleep | 14 (16.1) | 8 (14.0) | 6 (20.0) | Abdominal Pain | 11 (12.6) | 8 (14.0) | 3 (10.0) |
| Abdominal Pain | 12 (13.8) | 9 (15.8) | 3 (10.0) | Anorgasmia | 11 (12.6) | 8 (14.0) | 3 (10.0) |
| Anorgasmia | 11 (12.6) | 8 (14.0) | 3 (10.0) | Constipation | 10 (11.5) | 6 (10.5) | 4 (13.3) |
| Constipation | 11 (12.6) | 6 (10.5) | 5 (16.7) | Increased Sleep | 10 (11.5) | 6 (10.5) | 4 (13.3) |
| Muscle Contraction | 10 (11.5) | 6 (10.5) | 4 (13.3) | Muscle Contraction | 7 (8.0) | 3 (5.3) | 4 (13.3) |
| Blurred Vision | 9 (10.3) | 3 (5.3) | 6 (20.0) | Blurred Vision | 6 (6.9) | 2 (3.5) | 4 (13.3) |
| Weight Loss | 9 (10.3) | 5 (8.8) | 4 (13.3) | Tremor | 6 (6.9) | 3 (5.3) | 3 (10.0) |
| Increased Libido | 8 (9.2) | 6 (10.5) | 2 (6.7) | Delayed Ejaculation | 5 (5.7) | 3 (5.3) | 2 (6.7) |
| Tremor | 7 (8.0) | 4 (7.0) | 3 (10.0) | Edema | 4 (4.6) | 1 (1.8) | 3 (10.0) |
| Flushing | 6 (6.9) | 4 (7.0) | 2 (6.7) | Flushing | 3 (3.4) | 2 (3.5) | 1 (3.3) |
| Delayed Ejaculation | 6 (6.9) | 4 (7.0) | 2 (6.7) | Weight Loss | 3 (3.4) | 1 (1.8) | 2 (6.7) |
| Edema | 4 (4.6) | 1 (1.8) | 3 (10.0) | Increased Libido | 2 (2.3) | 1 (1.8) | 1 (3.3) |
| Erectile Dysfunction | 2 (2.3) | 1 (1.8) | 1 (3.3) | Erectile Dysfunction | 2 (2.3) | 1 (1.8) | 1 (3.3) |
| Premature Ejaculation | 0 (0.0) | 0 (0.0) | 0 (0.0) | Premature Ejaculation | 0 (0.0) | 0 (0.0) | 0 (0.0) |

Abbreviations: AD = Anxious Depression

**Table 5.** Summary of Weeks 10 to 12 Frequency and Severity of Side Effects in Aripiprazole Adjunctive Therapy Cohort (n = 84)^a^

| **Weeks 10 to 12 Frequency – Have Symptoms**  **n (%)** | **Overall**  **(n = 84)** | **No AD**  **(n = 55)** | **AD**  **(n = 29)** | **Weeks 10 to 12**  **Severity –**  **Have Trouble**  **n (%)** | **Overall**  **(n = 84)** | **No AD**  **(n = 55)** | **AD**  **(n = 29)** |
| --- | --- | --- | --- | --- | --- | --- | --- |
| Agitation | 34 (40.5) | 19 (34.5) | 15 (51.7) | Agitation | 29 (34.5) | 15 (27.3) | 14 (48.3) |
| Drowsiness | 33 (39.3) | 20 (36.4) | 13 (44.8) | Drowsiness | 28 (33.3) | 17 (30.9) | 11 (37.9) |
| Decreased Sleep | 29 (34.5) | 18 (32.7) | 11 (37.9) | Decreased Sleep | 26 (31.0) | 16 (29.1) | 10 (34.5) |
| Nervousness | 27 (32.1) | 16 (29.1) | 11 (37.9) | Weakness Fatigue | 25 (29.8) | 15 (27.3) | 10 (34.5) |
| Weakness Fatigue | 26 (31.0) | 16 (29.1) | 10 (34.5) | Nervousness | 22 (26.2) | 13 (23.6) | 9 (31.0) |
| Dry Mouth | 25 (29.8) | 14 (25.5) | 11 (37.9) | Dry Mouth | 20 (23.8) | 11 (20.0) | 9 (31.0) |
| Weight Gain | 23 (27.4) | 16 (29.1) | 7 (24.1) | Postural Hypotension | 19 (22.6) | 12 (21.8) | 7 (24.1) |
| Sweating | 21 (25.0) | 14 (25.5) | 7 (24.1) | Decreased Libido | 18 (21.4) | 10 (18.2) | 8 (27.6) |
| Postural Hypotension | 21 (25.0) | 13 (23.6) | 8 (27.6) | Weight Gain | 18 (21.4) | 14 (25.5) | 4 (13.8) |
| Decreased Libido | 20 (23.8) | 11 (20.0) | 9 (31.0) | Sweating | 17 (20.2) | 11 (20.0) | 6 (20.7) |
| Headache | 17 (20.2) | 11 (20.0) | 6 (20.7) | Decreased Appetite | 14 (16.7) | 8 (14.5) | 6 (20.7) |
| Decreased Appetite | 16 (19.0) | 10 (18.2) | 6 (20.7) | Diarrhea | 14 (16.7) | 9 (16.4) | 5 (17.2) |
| Diarrhea | 16 (19.0) | 10 (18.2) | 6 (20.7) | Headache | 14 (16.7) | 9 (16.4) | 5 (17.2) |
| Increased Appetite | 16 (19.0) | 11 (20.0) | 5 (17.2) | Dizziness | 13 (15.5) | 7 (12.7) | 6 (20.7) |
| Dizziness | 14 (16.7) | 8 (14.5) | 6 (20.7) | Increased Appetite | 13 (15.5) | 8 (14.5) | 5 (17.2) |
| Blurred Vision | 14 (16.7) | 9 (16.4) | 5 (17.2) | Dyspepsia | 12 (14.3) | 9 (16.4) | 3 (10.3) |
| Dyspepsia | 13 (15.5) | 10 (18.2) | 3 (10.3) | Increased Sleep | 12 (14.3) | 8 (14.5) | 4 (13.8) |
| Increased Sleep | 13 (15.5) | 9 (16.4) | 4 (13.8) | Blurred Vision | 11 (13.1) | 7 (12.7) | 4 (13.8) |
| Constipation | 12 (14.3) | 8 (14.5) | 4 (13.8) | Constipation | 11 (13.1) | 7 (12.7) | 4 (13.8) |
| Abdominal Pain | 11 (13.1) | 8 (14.5) | 3 (10.3) | Nausea | 10 (11.9) | 6 (10.9) | 4 (13.8) |
| Tremor | 11 (13.1) | 7 (12.7) | 4 (13.8) | Abdominal Pain | 8 (9.5) | 6 (10.9) | 2 (6.9) |
| Nausea | 10 (11.9) | 6 (10.9) | 4 (13.8) | Anorgasmia | 7 (8.3) | 3 (5.5) | 4 (13.8) |
| Muscle Contraction | 9 (10.7) | 6 (10.9) | 3 (10.3) | Tremor | 7 (8.3) | 3 (5.5) | 4 (13.8) |
| Flushing | 9 (10.7) | 7 (12.7) | 2 (6.9) | Muscle Contraction | 7 (8.3) | 4 (7.3) | 3 (10.3) |
| Anorgasmia | 8 (9.5) | 4 (7.3) | 4 (13.8) | Edema | 7 (8.3) | 3 (5.5) | 4 (13.8) |
| Delayed Ejaculation | 7 (8.3) | 5 (9.1) | 2 (6.9) | Flushing | 6 (7.1) | 4 (7.3) | 2 (6.9) |
| Edema | 6 (7.1) | 2 (3.6) | 4 (13.8) | Delayed Ejaculation | 5 (6.0) | 3 (5.5) | 2 (6.9) |
| Weight Loss | 6 (7.1) | 1 (1.8) | 5 (17.2) | Increased Libido | 3 (3.6) | 1 (1.8) | 2 (6.9) |
| Increased Libido | 5 (6.0) | 3 (5.5) | 2 (6.9) | Weight Loss | 2 (2.4) | 0 (0.0) | 2 (6.9) |
| Erectile Dysfunction | 1 (1.2) | 0 (0.0) | 1 (3.4) | Erectile Dysfunction | 1 (1.2) | 0 (0.0) | 1 (3.4) |
| Premature Ejaculation | 1 (1.2) | 0 (0.0) | 1 (3.4) | Premature Ejaculation | 1 (1.2) | 0 (0.0) | 1 (3.4) |

Abbreviations: AD = Anxious Depression

**Table 6.** Summary of Weeks 8 to 10 Frequency and Severity of Side Effects in Escitalopram Monotherapy Therapy Cohort (n = 79)^a^

| **Week 8 to 10 Symptoms Frequency – Have Symptoms**  **n (%)** | **Overall**  **(n = 79)** | **No AD**  **(n = 71)** | **AD**  **(n = 8)** | **Week 8 to 10 Symptoms Severity – Have Trouble**  **n (%)** | **Overall**  **(n = 79)** | **No AD**  **(n = 71)** | **AD**  **(n = 8)** |
| --- | --- | --- | --- | --- | --- | --- | --- |
| Drowsiness | 24 (30.4) | 22 (31.0) | 2 (25.0) | Drowsiness | 21 (26.6) | 20 (28.2) | 1 (12.5) |
| Sweating | 20 (25.3) | 17 (23.9) | 3 (37.5) | Dry Mouth | 16 (20.3) | 13 (18.3) | 3 (37.5) |
| Weakness Fatigue | 18 (22.8) | 15 (21.1) | 3 (37.5) | Weakness Fatigue | 16 (20.3) | 13 (18.3) | 3 (37.5) |
| Dry Mouth | 16 (20.3) | 13 (18.3) | 3 (37.5) | Decreased Libido | 15 (19.0) | 12 (16.9) | 3 (37.5) |
| Decreased Libido | 16 (20.3) | 13 (18.3) | 3 (37.5) | Sweating | 15 (19.0) | 12 (16.9) | 3 (37.5) |
| Agitation | 15 (19.0) | 10 (14.1) | 5 (62.5) | Agitation | 12 (15.2) | 9 (12.7) | 3 (37.5) |
| Nervousness | 14 (17.7) | 9 (12.7) | 5 (62.5) | Nervousness | 12 (15.2) | 8 (11.3) | 4 (50.0) |
| Anorgasmia | 13 (16.5) | 13 (18.3) | 0 (0.0) | Anorgasmia | 11 (13.9) | 11 (15.5) | 0 (0.0) |
| Decreased Appetite | 13 (16.5) | 10 (14.1) | 3 (37.5) | Decreased Sleep | 11 (13.9) | 9 (12.7) | 2 (25.0) |
| Postural Hypotension | 13 (16.5) | 11 (15.5) | 2 (25.0) | Weight Gain | 11 (13.9) | 8 (11.3) | 3 (37.5) |
| Decreased Sleep | 12 (15.2) | 10 (14.1) | 2 (25.0) | Headache | 11 (13.9) | 10 (14.1) | 1 (12.5) |
| Weight Gain | 12 (15.2) | 9 (12.7) | 3 (37.5) | Increased Appetite | 11 (13.9) | 9 (12.7) | 2 (25.0) |
| Headache | 12 (15.2) | 11 (15.5) | 1 (12.5) | Dizziness | 10 (12.7) | 7 (9.9) | 3 (37.5) |
| Dizziness | 11 (13.9) | 8 (11.3) | 3 (37.5) | Increased Sleep | 10 (12.7) | 8 (11.3) | 2 (25.0) |
| Increased Appetite | 11 (13.9) | 9 (12.7) | 2 (25.0) | Decreased Appetite | 9 (11.4) | 7 (9.9) | 2 (25.0) |
| Increased Sleep | 11 (13.9) | 9 (12.7) | 2 (25.0) | Postural Hypotension | 9 (11.4) | 8 (11.3) | 1 (12.5) |
| Weight Loss | 10 (12.7) | 9 (12.7) | 1 (12.5) | Dyspepsia | 7 (8.9) | 7 (9.9) | 0 (0.0) |
| Dyspepsia | 8 (10.1) | 8 (11.3) | 0 (0.0) | Diarrhea | 7 (8.9) | 6 (8.5) | 1 (12.5) |
| Diarrhea | 8 (10.1) | 7 (9.9) | 1 (12.5) | Delayed Ejaculation | 7 (8.9) | 6 (8.5) | 1 (12.5) |
| Muscle Contraction | 8 (10.1) | 6 (8.5) | 2 (25.0) | Nausea | 6 (7.6) | 6 (8.5) | 0 (0.0) |
| Nausea | 8 (10.1) | 8 (11.3) | 0 (0.0) | Tremor | 4 (5.1) | 3 (4.2) | 1 (12.5) |
| Delayed Ejaculation | 7 (8.9) | 6 (8.5) | 1 (12.5) | Abdominal Pain | 3 (3.8) | 2 (2.8) | 1 (12.5) |
| Flushing | 5 (6.3) | 3 (4.2) | 2 (25.0) | Muscle Contraction | 3 (3.8) | 3 (4.2) | 0 (0.0) |
| Abdominal Pain | 4 (5.1) | 3 (4.2) | 1 (12.5) | Flushing | 3 (3.8) | 2 (2.8) | 1 (12.5) |
| Tremor | 4 (5.1) | 3 (4.2) | 1 (12.5) | Blurred Vision | 2 (2.5) | 1 (1.4) | 1 (12.5) |
| Constipation | 3 (3.8) | 2 (2.8) | 1 (12.5) | Constipation | 2 (2.5) | 2 (2.8) | 0 (0.0) |
| Increased Libido | 3 (3.8) | 3 (4.2) | 0 (0.0) | Weight Loss | 2 (2.5) | 2 (2.8) | 0 (0.0) |
| Blurred Vision | 2 (2.5) | 1 (1.4) | 1 (12.5) | Increased Libido | 1 (1.3) | 1 (1.4) | 0 (0.0) |
| Edema | 2 (2.5) | 1 (1.4) | 1 (12.5) | Erectile Dysfunction | 1 (1.3) | 1 (1.4) | 0 (0.0) |
| Erectile Dysfunction | 1 (1.3) | 1 (1.4) | 0 (0.0) | Edema | 0 (0.0) | 0 (0.0) | 0 (0.0) |
| Premature Ejaculation | 0 (0.0) | 0 (0.0) | 0 (0.0) | Premature Ejaculation | 0 (0.0) | 0 (0.0) | 0 (0.0) |

Abbreviations: AD = Anxious Depression

**Table 7.** Summary of Weeks 10 to 12 Frequency and Severity of Side Effects in Escitalopram Monotherapy Therapy Cohort (n = 80)^a^

| **Week 10 to 12 Symptoms Frequency – Have Symptoms**  **n (%)** | **Overall**  **(n = 80)** | **No AD**  **(n = 72)** | **AD**  **(n = 8)** | **Week 10 to 12 Symptoms Severity – Have Trouble**  **n (%)** | **Overall**  **(n = 80)** | **No AD**  **(n = 72)** | **AD**  **(n = 8)** |
| --- | --- | --- | --- | --- | --- | --- | --- |
| Drowsiness | 29 (36.2) | 26 (36.1) | 3 (37.5) | Drowsiness | 27 (33.8) | 24 (33.3) | 3 (37.5) |
| Sweating | 18 (22.5) | 15 (20.8) | 3 (37.5) | Sweating | 16 (20.0) | 13 (18.1) | 3 (37.5) |
| Nervousness | 17 (21.2) | 12 (16.7) | 5 (62.5) | Weakness Fatigue | 16 (20.0) | 12 (16.7) | 4 (50.0) |
| Weakness Fatigue | 17 (21.2) | 13 (18.1) | 4 (50.0) | Dry Mouth | 15 (18.8) | 13 (18.1) | 2 (25.0) |
| Decreased Libido | 16 (20.0) | 13 (18.1) | 3 (37.5) | Decreased Libido | 14 (17.5) | 11 (15.3) | 3 (37.5) |
| Dry Mouth | 15 (18.8) | 13 (18.1) | 2 (25.0) | Anorgasmia | 13 (16.2) | 13 (18.1) | 0 (0.0) |
| Decreased Sleep | 15 (18.8) | 13 (18.1) | 2 (25.0) | Decreased Sleep | 13 (16.2) | 11 (15.3) | 2 (25.0) |
| Agitation | 14 (17.5) | 10 (13.9) | 4 (50.0) | Headache | 13 (16.2) | 9 (12.5) | 4 (50.0) |
| Headache | 14 (17.5) | 10 (13.9) | 4 (50.0) | Nervousness | 13 (16.2) | 8 (11.1) | 5 (62.5) |
| Anorgasmia | 13 (16.2) | 13 (18.1) | 0 (0.0) | Agitation | 12 (15.0) | 8 (11.1) | 4 (50.0) |
| Diarrhea | 13 (16.2) | 11 (15.3) | 2 (25.0) | Diarrhea | 12 (15.0) | 10 (13.9) | 2 (25.0) |
| Increased Appetite | 13 (16.2) | 12 (16.7) | 1 (12.5) | Increased Sleep | 12 (15.0) | 10 (13.9) | 2 (25.0) |
| Increased Sleep | 12 (15.0) | 10 (13.9) | 2 (25.0) | Delayed Ejaculation | 9 (11.2) | 8 (11.1) | 1 (12.5) |
| Weight Gain | 10 (12.5) | 9 (12.5) | 1 (12.5) | Dizziness | 8 (10.0) | 7 (9.7) | 1 (12.5) |
| Nausea | 10 (12.5) | 9 (12.5) | 1 (12.5) | Increased Appetite | 8 (10.0) | 7 (9.7) | 1 (12.5) |
| Postural Hypotension | 10 (12.5) | 10 (13.9) | 0 (0.0) | Weight Gain | 7 (8.8) | 6 (8.3) | 1 (12.5) |
| Delayed Ejaculation | 10 (12.5) | 9 (12.5) | 1 (12.5) | Nausea | 7 (8.8) | 6 (8.3) | 1 (12.5) |
| Weight Loss | 9 (11.2) | 8 (11.1) | 1 (12.5) | Postural Hypotension | 7 (8.8) | 7 (9.7) | 0 (0.0) |
| Dyspepsia | 8 (10.0) | 6 (8.3) | 2 (25.0) | Dyspepsia | 6 (7.5) | 4 (5.6) | 2 (25.0) |
| Dizziness | 8 (10.0) | 7 (9.7) | 1 (12.5) | Abdominal Pain | 5 (6.2) | 4 (5.6) | 1 (12.5) |
| Muscle Contraction | 7 (8.8) | 6 (8.3) | 1 (12.5) | Flushing | 5 (6.2) | 4 (5.6) | 1 (12.5) |
| Increased Libido | 7 (8.8) | 6 (8.3) | 1 (12.5) | Constipation | 4 (5.0) | 4 (5.6) | 0 (0.0) |
| Abdominal Pain | 6 (7.5) | 5 (6.9) | 1 (12.5) | Decreased Appetite | 4 (5.0) | 3 (4.2) | 1 (12.5) |
| Tremor | 6 (7.5) | 4 (5.6) | 2 (25.0) | Tremor | 4 (5.0) | 3 (4.2) | 1 (12.5) |
| Flushing | 6 (7.5) | 4 (5.6) | 2 (25.0) | Muscle Contraction | 4 (5.0) | 3 (4.2) | 1 (12.5) |
| Constipation | 5 (6.2) | 4 (5.6) | 1 (12.5) | Blurred Vision | 3 (3.8) | 2 (2.8) | 1 (12.5) |
| Decreased Appetite | 5 (6.2) | 3 (4.2) | 2 (25.0) | Increased Libido | 2 (2.5) | 1 (1.4) | 1 (12.5) |
| Blurred Vision | 4 (5.0) | 3 (4.2) | 1 (12.5) | Weight Loss | 2 (2.5) | 2 (2.8) | 0 (0.0) |
| Edema | 1 (1.2) | 1 (1.4) | 0 (0.0) | Edema | 1 (1.2) | 1 (1.4) | 0 (0.0) |
| Erectile Dysfunction | 1 (1.2) | 1 (1.4) | 0 (0.0) | Erectile Dysfunction | 1 (1.2) | 1 (1.4) | 0 (0.0) |
| Premature Ejaculation | 0 (0.0) | 0 (0.0) | 0 (0.0) | Premature Ejaculation | 0 (0.0) | 0 (0.0) | 0 (0.0) |

Abbreviations: AD = Anxious Depression
